# A Hierarchy-Based System for Recognizing Customer Activity in Retail Environments

**DOI:** 10.3390/s21144712

**Published:** 2021-07-09

**Authors:** Jiahao Wen, Luis Guillen, Toru Abe, Takuo Suganuma

**Affiliations:** 1 Graduate School of Information Sciences, Tohoku University, 2-1-1 Katahira, Aoba-ku, Sendai 980-8577, Japan; jh-wen@ci.cc.tohoku.ac.jp; 2 Research Institute of Electrical Communication, Tohoku University, 2-1-1 Katahira, Aoba-ku, Sendai 980-8577, Japan; lguillen@ci.cc.tohoku.ac.jp; 3 Cyberscience Center, Tohoku University, 2-1-1 Katahira, Aoba-ku, Sendai 980-8577, Japan; suganuma@tohoku.ac.jp

**Keywords:** retail environment, customer activity, activity hierarchy, activity recognition

## Abstract

Customer activity (CA) in retail environments, which ranges over various shopper situations in store spaces, provides valuable information for store management and marketing planning. Several systems have been proposed for customer activity recognition (CAR) from in-store camera videos, and most of them use machine learning based end-to-end (E2E) CAR models, due to their remarkable performance. Usually, such E2E models are trained for target conditions (i.e., particular CA types in specific store spaces). Accordingly, the existing systems are not malleable to fit the changes in target conditions because they require entire retraining of their specialized E2E models and concurrent use of additional E2E models for new target conditions. This paper proposes a novel CAR system based on a hierarchy that organizes CA types into different levels of abstraction from lowest to highest. The proposed system consists of multiple CAR models, each of which performs CAR tasks that belong to a certain level of the hierarchy on the lower level’s output, and thus conducts CAR for videos through the models level by level. Since these models are separated, this system can deal efficiently with the changes in target conditions by modifying some models individually. Experimental results show the effectiveness of the proposed system in adapting to different target conditions.

## 1. Introduction

Customer activity (CA) in retail environments, which ranges over various shopper situations in store spaces, provides valuable information for store management (e.g., layout optimization and shoplifting prevention) and marketing planning (e.g., supply control and product development) [1,2]. However, in traditional retail environments, available information tends to be limited to purchase records of customers, which cannot reveal any details of CA such as movement of customers in store spaces and interaction of customers with products [3]. Therefore, several systems have been proposed for customer activity recognition (CAR) from videos taken by in-store cameras, and their efforts are devoted to customer detection, counting, re-identification, tracking, behavior recognition, and so on [4].

**Figure 1:**
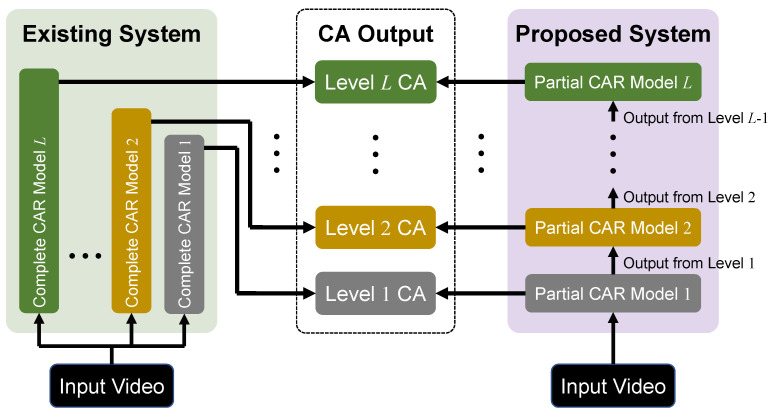
Structures of a typical existing CAR system and our proposed CAR system.

Most of the existing CAR systems use machine learning based end-to-end (E2E) models due to their remarkable accuracy in CAR [4]. Usually such E2E models in CAR systems are constructed as complete CAR models, each of which is trained to recognize particular types of CA from input videos of specific store spaces. Accordingly, when target conditions (i.e., CA types and store spaces) change, these systems are not flexible enough deal with the changes because they require new training data and full retraining of their CAR models for new conditions. In addition to this, since each CAR model is mostly built to recognize the CA types at a certain level (e.g., customer positions, movements, behavior), if CA types at different levels are needed simultaneously, as shown in Figure 1, the concurrent use of additional complete CAR models is required. Since those models share the same input video, the concurrent use of the models brings the redundancy of low-level processing, such as detecting object regions in the input video. The redundant processing result in the low efficiency of the CAR system. Moreover, the same input also leads to similar low-level processing, which forms a tightly-coupled structure with the concurrent use of several complete CAR models. The tightly-coupled structure causes low maintainability in a CAR system. The target CA types and levels vary widely depending on purposes of store management, goals of marketing planning, phases in development work, etc. Furthermore, the target store spaces look different depending on product display, shelf arrangement, or in-store camera layout. Consequently, flexibility to deal efficiently with the changes in the target conditions is one of the most important issues for CAR systems.

As the objective is to design a CAR system that has good efficiency, flexibility, and maintainability to provide various types of CA to fit different target conditions, we summarize the output CA in existing CAR systems or methods. In this paper, we propose a novel CAR system based on a hierarchy that organizes CA types into different levels of abstraction from lowest to highest. As shown in Figure 1, the proposed system is composed of multiple partial CAR models, each of which corresponds to the recognition process of a certain CA level and performs the CAR process on the lower level output. Thus, CAR from videos is conducted through the partial CAR models level by level, without redundant processing. Since the partial CAR models in the proposed CAR system are separated and layered by CA levels, these loosely-coupled CAR models can be replaced or modified individually. As a result, the proposed CAR system has the following advantages over the existing CAR systems:

1. CA output at different levels can be simultaneously provided by the proposed system as the output from different partial CAR models compared to a single complete CAR model. With the avoidance of redundant low-level processing, the proposed CAR system tends to be efficient;
2. The changes in target CA types and store spaces can be dealt with easily by modifying some partial CAR models in the proposed system individually, which shows better flexibility;
3. Maintainability of the proposed system is increased because each partial CAR model can be updated independently.

In the evaluation part, we evaluate both existing CAR system and our proposed CAR system on their running performance (accuracy, speed, etc.), flexibility, and maintainability. Results show that the proposed CAR system is feasible to conduct CAR tasks and outperforms the existing CAR system in flexibility and maintainability with the increase in the number of complete CAR models in the existing CAR system. It indicates that the proposed CAR system can fit different target conditions.

The remainder of this paper is organized as follows: Section 2 provides an overview of the existing CAR systems. Section 3 describes our proposal of the hierarchy-based CAR system. In Section 4, a comparative evaluation of the proposed CAR system with the existing E2E model-based CAR systems. Finally, Section 5 concludes this paper with some final remarks and future work.

## 2. Related Work

As the objective is a CAR system that can provide various types of CA to fit different target conditions, we should summarize the output CA in existing CAR systems or methods. Therefore, this section focus on the type of output CA in existing research first. Then, the disadvantages of existing CAR systems or methods are discussed.

### 2.1. Various Types of CA

Till now, existing researches provide lots of ways to get various types of CA. In Table 1, we summarize those CA into three categories, object location, object movement, and customer behavior.

#### 2.1.1. Object Location

In retail environments, an object might refer to, among others, humans, products, shopping carts. Researches that provide the information of an object’s location belong to this category. Most CAR researches focus on the human detection. Different sensors are applied to detect humans [2], in particular, WiFi RSS [5,6,7], RFID [11], GPS [12], Bluetooth beacons [8,9,10], RGB camera [3,13,14,20,21,26], RGB-depth camera [4,15,16,17,18,19]. Due to its descriptive nature, visual data are the preferred input for human detection. Nevertheless, few researches [18] combine multiple sensors. Conventional methods detect humans in data extracted from RGB images by using a histogram of oriented gradients (HOG) [13,14]. However, more recently, machine-learning tools for human detection, especially using convolutional neural network (CNN) [3,21,26] were developed because of their excellent performance concerning the detection speed and accuracy in a wide range of environments.

Compared to RGB-based-only methods, the combination of top-view and RGB-depth cameras provides more straightforward solutions for human detection [27]. The top-view image offers an occlusion-free view as the occlusion rarely occurs in the top-view direction, which takes images from directly above. It also preserves privacy since faces are usually not exposed to the camera. As the depth values change significantly in the human region, the legacy human detection method [15,16,19] is to run background subtraction on the top-view RGB-D images. Another method [17] uses self-designed features transformed from the original RGB-D pixels to detect humans. Additionally, though most methods detect the whole region of the human body, some approaches [1,3,4,21] also focus on some specific body parts, such as the head and hand. Additionally, except for human detection, Merad et al. estimate behavior by detecting products and hand gestures from the images of a wearable device. Additionally, Zhao et al. [23] proposed a combined neural network-based model to detect products from RGB images. Finally, Paolanti et al. [22] detect humans by locating shopping carts and baskets with ultra-wideband (UWB) technology.

#### 2.1.2. Object Movement

Movement reveals how an object moves in a time span. We consider the CAR researches that output object tracking results as belonging to this category. Similar to the detection tasks, more efforts have been devoted to processing visual data for tracking tasks. The tracking target tends to be humans rather than other objects [22,23]. Some methods track objects for a further purpose, such as behavior recognition. Therefore, the object’s coordinates in each frame are unnecessary. They track pixels, for instance, optical flow [24,25]. Instead of tracking pixels, most methods track the object’s coordinates. Kalman filter [17,19] and particle filter [3] are common solutions to the coordinate tracking tasks. Some approaches also combine both filters [18] to achieve better tracking results. In some cases of a reliable detector, the intersection over union (IoU) tracker is also applied [15,16].

#### 2.1.3. Customer Behavior

Behavior recognition is always a challenging topic [28] due to the methodology involved in features, such as the inter-, intra-class variations, cluttered background, or changes of the camera views. In the context of retail, existing methods mainly share the pattern of extracting the spatial features from consecutive frames and recognizing behavior from the sequenced spatial features by models, especially the hidden Markov model (HMM). Popa et al. [24,25] proposed an HMM-based model to recognize customer behavior with optical flow features. Singh et al. [26] use CNN to extract spatial features of each video frame and recognize behavior by long-short term memory (LSTM) [29] with the spatial features. Compared to the pixel level features, some approaches utilize HMM [1], dynamic Bayesian network [3], and support vector machine (SVM) [19] to analyze coordinate trajectory for behavior recognition. In addition, due to the property of some customer behavior, some methods designed self-defined rule-based models [4,15,16] to recognize behavior.

Concerning the output CA, namely the recognized customer behavior, current approaches provide the recognition of various customer behavior. Some common customer-product interactions, such as “pick a product”, “return a product”, “holding a product”, are recognized in most approaches. Other models also observe indirect behavior like “pass by”, “viewing the product shelf”. Additionally, some customer behavior [19] are excluded because of their misleading definition, and some are merged due to similar definitions. In some particular cases, Popa et al. [25] recognize customer’s interactions with clothes in a clothing store. Despite the variety of recognition results, few researchers comprehensively categorize customer behavior. Popa et al. [25] extract spatial and temporal features with histograms of optical flow (HOF). In this research, the behavior is divided by the level of granularity of HOF. Besides, Generosi et al. [21] estimate customer’s emotion, which is also a type of customer behavior, with facial expressions and speech text. However, this feature is out of the scope of this paper, as might breach the privacy of customers.

### 2.2. Existing CAR Methods and Systems

About CAR tasks, existing researches tend to focus on a particular CAR method for one type of CAR task instead of a CAR system for several types of CAR tasks. However, a single CAR method has limited types of output CA. The usage of E2E models makes it difficult to adapt to any change of target conditions. Therefore, a CAR system is necessary to get various types of CA and fit the changes of target conditions. Nevertheless, the researches for a particular CAR task do not provide a view of the common CAR system. In few papers that implement a CAR system, they share the common structure as shown in Figure 1. The complete CAR model refers to a single CAR method for one type of CA. Different complete CAR models are concurrently used to get various types of CA. Since the wide usage of E2E machine learning-based CAR models, such a system is named “E2E model-based CAR system”.

As described above, some complete CAR models detect and track objects for further behavior recognition. However, those models start the processing from video frames instead of utilizing the results of state-of-the-art detection and tracking models. From all the authors, only Popa et al. [24] start from trajectory analysis with given detection annotations. Thus, to get different types of CA simultaneously, several complete CAR models should be concurrently used, which means redundant similar computation on object detection and tracking. Consequently, E2E model-based CAR system tends to have low efficiency and a tightly-coupled structure. Due to the existence of redundant similar processing in different CAR models and the use of pre-trained models, once the environment (i.e., product appearance, camera view, store layout) is changed, existing systems can only be updated entirely because all CAR models are related to this change. More than any of these, the use of pre-trained models makes it hard to adapt to the target changes as those changes require time-consuming work of collecting new data and retraining models. Thus, the heavy workload to fit those changes results in the low flexibility of E2E model-based CAR system. Additionally, with the use of complete CAR models, the whole CAR system can only be updated by models instead of process. In other words, it is difficult to update any process of a complete CAR model, such as the part of human detection of a complete behavior recognition model. Therefore, the E2E model-based CAR systems have low maintainability.

## 3. Hierarchy-Based CAR System

**Figure 2:**
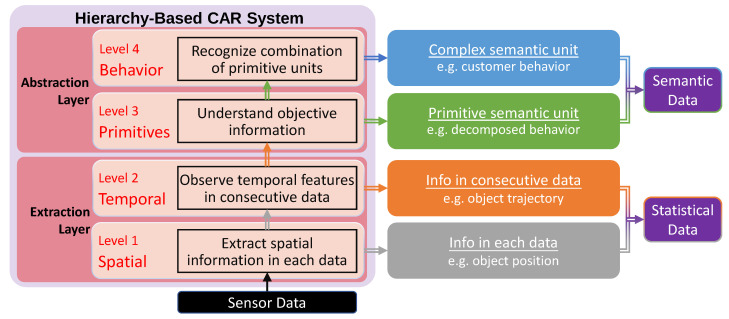
Structure of our proposed hierarchy-based CAR system.

This section gives an overview of the proposed hierarchy-based system and explains the details of every hierarchy level. The structure of the system is depicted in Figure 2 where the sensor data refers to unprocessed data from various sensors, which is input into the hierarchy for further processing.

### 3.1. CAR Hierarchy Overview

The hierarchy comprises four levels: spatial, temporal, primitives, and behavior, where spatial and temporal belong to the extraction layer, primitives and behavior belong to the abstraction layer. The output of each layer refers to a type of CA. Therefore, the hierarchy could provide various CA to satisfy different target conditions. Compared to the contrived categories of existing types of CA, a new level primitives is added for malleable customer behavior recognition. In the hierarchy, objective information is extracted from sensor data at first. Then, the objective information is abstracted as semantic units, such as customer behavior. As observed, spatial is the bottom level of the hierarchy, which extracts the spatial information in each sensor data, such as an object’s position in each frame. Then, level temporal observes temporal features of the spatial outputs, for instance, getting an object’s trajectory from consecutive position results. In the upper level, primitives, the system abstracts the primitive semantic units, which is called an “event” in this paper, from the temporal outputs. In this paper, an event is considered the summary of a trajectory that has similar spatial and temporal features. Hence, this output is a semantically primitive feature. Finally, level behavior recognizes customer behavior by matching different combinations of events. As the behavior consists of events, it is regarded as complex semantic unit, which means the behavior output can be decomposed into events.

### 3.2. Level 1: Spatial

As the name implies, this level conducts detection methods with the sensor data. Generally, all sensors have a frequency of collecting data, such as the frame per second (FPS) for cameras, the sampling rate for sensors that transmit electromagnetic signals. Level spatial is designed for the spatial information extraction of each sampled sensor data. Commonly, this level detects objects’ position in each camera frame. In the case of other sensors, they also locate objects in a specific coordinate system. Since this paper proposes a hierarchical design, the methods in every level are not expected to be limited to a particular method. Any detection method can be implemented into this level as long as it is qualified to receive sensor data as input and output extracted spatial information of each sampled sensor data. Thus, all the object detection methods can be implemented into this level. Additionally, multiple methods are allowed to be implemented to get more reliable results.

### 3.3. Level 2: Temporal

This level observes the temporal features of consecutive spatial results. The temporal features contain object-level features, such as trajectory, and pixel-level features, such as optical flow. The object-level method tracks the object’s trajectory with its position in each frame. The pixel-level method extracts pixel features from the change of pixels, such as the optical flow. The same as level spatial, Kalman filter [30] for tracking, optical flow estimation, or any other approach capable to perform this level’s process can be applied to this level.

### 3.4. Level 3: Primitives

This level is inspired by [31] that defines human behavior as a composition of multiple events. An event refers to a single low-level spatiotemporal entity that cannot be further decomposed. Hence, “event” refers to a primitive semantic unit in this paper. Since there are no available methods in the related work for this category, we proposed a novel method as follows. In the proposed method, the input is assumed to be the object’s coordinate trajectory, and the output includes motion event, which describes the motion of an object, and relation event, which describes the relation of two objects.

As the task of abstracting objective information should be done in primitives, we design two steps in the proposed method. The two steps summarize objective information and provide subjective abstractions of the objective information. Firstly, trajectory segmentation is carried out to separate a trajectory into several segments with similar features. Then, we symbolize each segment to make the numerical value into human-readable symbols.

#### 3.4.1. Trajectory Segmentation

Trajectory segmentation refers to the action of dividing the trajectory into several segments by key-points. The segments preserve most features of the original trajectory. Since a trajectory reveals the motion of an object, current segmentation approaches [32,33] separate a trajectory based on the moving distance and direction of each vector in the trajectory. Concerning the approximate trajectory partitioning (ATP) [32], we design an ATP-based algorithm for trajectory segmentation in the retail environment. Since ATP processes trajectory data from GPS-based navigation applications, it is sensitive to the direction changes even if the vector has a very short moving distance. However, if a trajectory is collected from cameras in retail environments, a small-length vector in the trajectory usually means the object is idling without any movement. In this case, the algorithm only reacts to the change of moving distance. Hence, we process ATP’s output by a thresholding algorithm in Algorithm 1. The algorithm receives two inputs: a list of key-points’ coordinate ATPKpts which is the results of ATP, a threshold $threshold_{0}$ to determine whether an object has stopped. It preserves the key-points with a moving distance longer than $threshold_{0}$. Namely, the segments with short moving distances are merged.
**Algorithm 1:** Thresholding algorithm for trajectory segmentation.
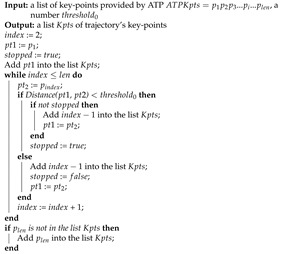


**Figure 3:**
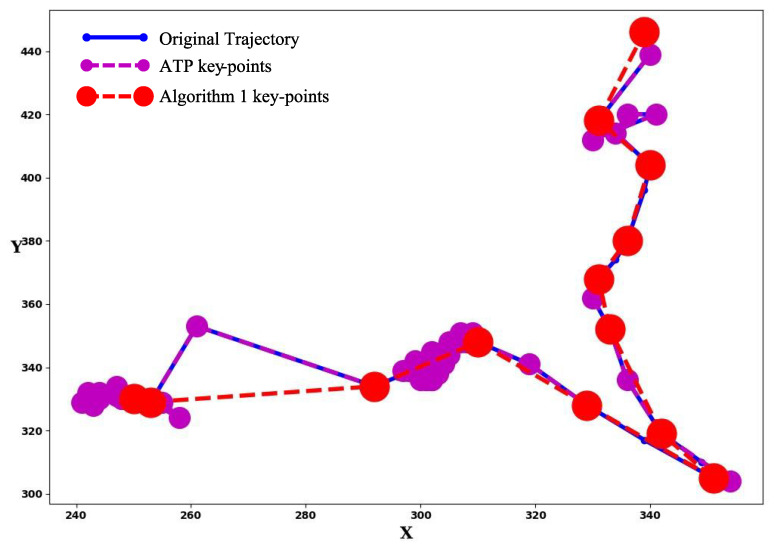
Example result of trajectory segmentation.

Figure 3 shows an example result of the trajectory segmentation algorithm. The blue and smallest points and solid lines represent the original trajectory. The larger purple points and dashed lines are the result key-points of ATP. As it is sensitive to the direction, nearly all the idling points are marked as key-points. The largest red points and dashed lines refer to the key-points processed by Algorithm 1. As observed, all the idling points are merged.

#### 3.4.2. Segment Symbolization

Although the segmentation results contain the subjective abstraction of the input trajectory, the numerical coordinate values in segments are not human-readable. Hence, we symbolize each segment in this step. The symbolization result is a four-dimension vector named “motion event” (ME). Algorithm 2 shows the symbolization process, as observed, the first input is a segment $S=[object,p_{start}p_{end}]$, where object is the object’s name of this segment and $p_{start}p_{end}$ refers to the start and end coordinates of the segment. The other inputs are parameters related to the output. The output is ME=[object,motion,start,end]. If the segment moves a distance smaller than $threshold_{1}$, motion is considered as “stop”. Otherwise, motion is determined by the segment’s direction. Therefore, motion describes an object’s move status. start and end represent the start and end point of the segment, respectively. However, the input $p_{start}p_{end}$ are numerical values. To symbolize the coordinate, we separate each video frame into several areas areas and assign a name for each area. Then, the algorithm checks the located area of the start and end point. Thus, the values of start and end are the corresponding areas’ names.

ME can describe the motion of an object. However, the customer behavior usually includes the interaction between several objects. Algorithm 3 shows the process to symbolize two segments into a four-dimension vector called “relation event” (RE), which describes the relation between two objects. The inputs are two segments $S^{1},S^{2}$ and two parameters $threshold_{2},threshold_{3}$ related to the output. If the distance from $S^{1}$ to $S^{2}$ is larger than $threshold_{2}$, the segments are regarded as “no relation” as they are too far away from each other. Otherwise, relation is determined by the distance of two segments’ start points diststart and end points distend. Therefore, relation describes how two objects move in regard to one another. relativePosition represents the relative position of $object^{1}$ compared to $object^{2}$, and it is determined by the direction of a segment’s end point to another one’s end point. With our proposed method for trajectory segmentation and symbolization, level primitives can extract the objective trajectory information and output human-readable primitive semantic units.
**Algorithm 2:** Symbolization: Motion Event.
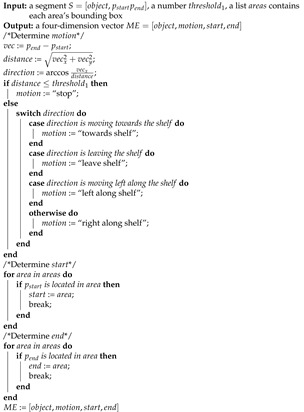


### 3.5. Level 4: Behavior

As the name implies, this level of the hierarchy performs the customer behavior recognition. Additionally, we propose a method for this level because of no available methods. Before the recognition, each behavior is predefined as permutations and combinations of several events called an “event pattern.” The input events are received in a sequence, which then forms a timeline that describes the chronological order of events. The behavior can be recognized once the event pattern is matched from the timeline.

In this paper, we design a table that formulates the definition of behavior by events. For instance, Table 2 defines the behavior “pick a product”. The column “Behavior” includes the behavior’s name. The column “Event Pattern” refers to the definition of the behavior. In this case, the definition “ME 1 → ME 2 & RE 3” can be explained as ME 1 happens at the beginning, then, ME 2 and RE 3 happen concurrently. Namely, the symbol “→” implies the events on its left-side happen first, and “&” means its adjacent events happen concurrently. At last, concerning the column “Content of Events”, it contains the content of events that are used in the definition. About the located area of ME 1 and ME 2, we divide each video frame into two areas. “SA” refers to the shelf area, which includes the shelf and product’s region in the frame. Another area is the viewing area (VA), which refers to the area that a person is close to the shelf enough to interact with the products on the shelf. With the columns of content and definition, the definition of “pick a product” can be explained in human language that a hand moves towards shelf, then, the hand leaves the shelf with product A following the hand.
**Algorithm 3:** Symbolization: Relation Event.
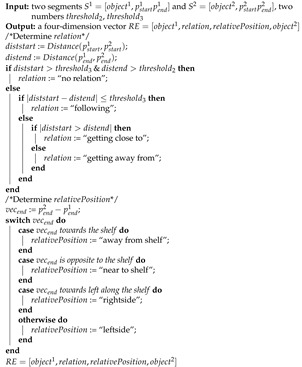


**Figure 4:**
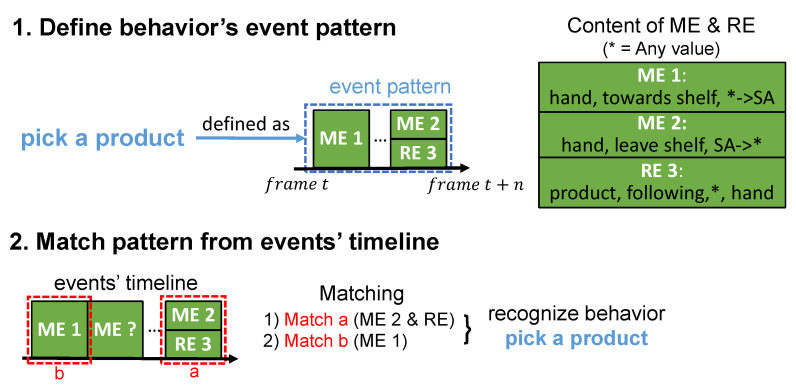
Process flow of the customer behavior recognition in level 4.

There are two steps in our proposed behavior recognition method as illustrated in Figure 4. First of all, we define behavior’s event pattern. Figure 4 depicts an example definition of “pick a product”, which is the same as Table 2. Each green block on the timeline refers to a motion event or a relation event. The blocks in blue dashed line represent the range of defined event pattern. Secondly, we match the pattern from event’s timeline. Once the pattern is matched in the event’s timeline, the behavior is recognized. In Figure 4, the matched events are figured out by blocks in red dashed line. As the event inputs from level 3 are usually dynamically changed, matching events along the timeline is computationally expensive. Thus, we match events along the timeline in reverse. In this case, block a is matched before block b, namely, the latest events are matched at first.

As customer behavior consists of several events, it is taken as complex semantic units in this paper. By changing the event pattern, the method is malleable enough to define a different customer behavior recognition without major structural change in the model. This makes it possible to adapt to changes in recognition targets. To sum up, our proposed CAR system organizes CAR tasks into a four-level hierarchy, which provides adaptability to serve different needs. Moreover, since similar CAR processes are separated by levels, redundant processes are avoided in such hierarchical structure, which is efficient. As the loosely-coupled design allows the partial CAR model in each level to be replaced individually, the proposed system could be better in maintainability.

## 4. Evaluation Experiments

This section evaluates the running performance, the flexibility, and the system structure of our proposed hierarchy-based CAR system compared to E2E model-based systems. Initially, we explain the implementation of both types of systems, the dataset used, and the metrics we used to measure their running performance, flexibility, and structure.

### 4.1. System Implementation

**Figure 5:**
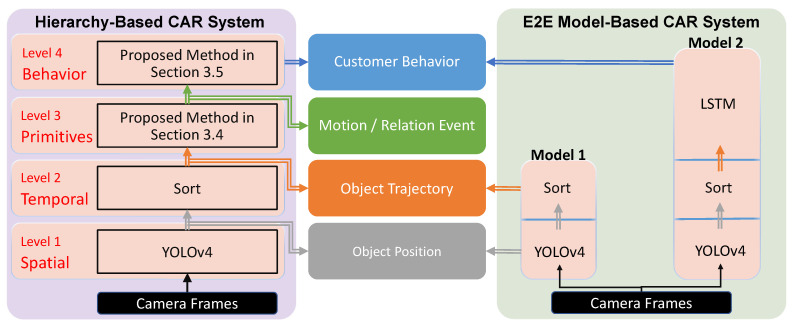
Structure and implementation of the proposed hierarchy-based system and existing E2E model-based system.

Figure 5 shows the structure and implemented methods of both systems, the proposed one and the E2E-model one. As the visual data contains more information than any other sensor data, camera frames are assumed as the input of both systems. For the hierarchy-based system, YOLOv4 [34] and Sort [35] are implemented into level spatial and temporal for object detection and tracking, respectively. In level event and behavior, we use our proposed methods, as described in Section 3.4 and Section 3.5. For the E2E model-based system, we build two models. Model 1 contains YOLOv4 and Sort for object detection and tracking. As there is no available method for event, the model that outputs events is not built. Model 2 is built for customer behavior recognition with LSTM [29]. Both systems are implemented on a desktop with Windows 10, 16 GB of RAM, Intel Core i7-9700 CPU 3.00 GHz, NVIDIA GeForce RTX 2060 GPU (6 GB).

### 4.2. Dataset

**Figure 6:**
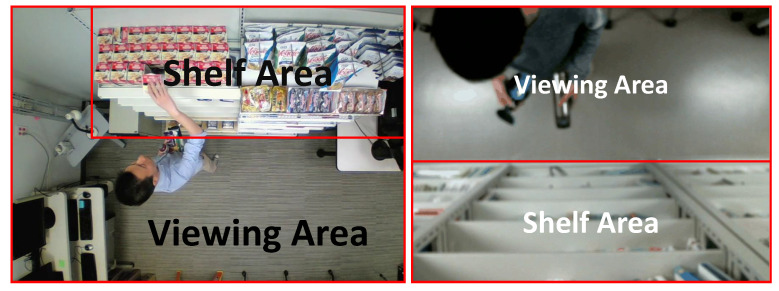
Example frame and area division of DS 1 (**left**) and DS 2 (**right**).

**Figure 7:**
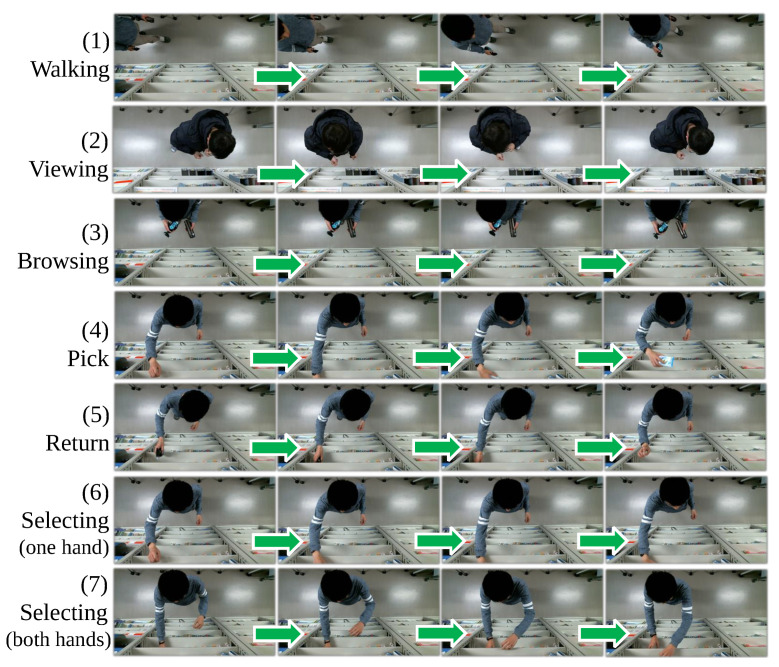
Example of behavior annotation in DS 2.

Two datasets are utilized in the experiments, one is MERL shopping dataset (DS 1) [26] that consists of 106 videos. Each video is about 2 min long with a resolution of 920 × 680 and an FPS of 30. A fixed RGB top-view camera is installed to observe people shopping in a retail environment. The left in Figure 6 shows an example frame of DS 1 and the area division for each frame. Each video contains the start and end frames of the behavior as annotations, types of behaviors are as following: reach to shelf (reach hand into shelf), retract from shelf (retract hand from shelf), hand in shelf (extended period with hand in the shelf), inspect product (inspect product while holding it in hand, the type of products is not identified), and inspect shelf (look at shelf while not touching or reaching for the shelf). The second dataset (DS 2) is collected on our own. It comprises 19 half-a-minute videos with a resolution of 480 × 640 and an FPS of 15. Similar to DS 1, we build a laboratory retail environment and install an RGB top-view camera to get an occlusion-free view. Figure 6 shows its example frame and area division. Videos are collected at a public activity, where the random 19 participants are requested to simulating shopping in front of the shelf one by one. Figure 7 shows the example behavior annotations of DS 2. The start and end frames of those behavior in the videos are annotated. Except for the behavior annotations, we also annotate the bounding box of body, hands, and four products in each frame for the training of level 1 and level 2. About the annotator, both behavior and bounding box are annotated by one of the authors. Additionally, due to the privacy issues, DS 2 is currently not publicly available.

### 4.3. Performance Evaluation

To compare the performance of both systems in dealing with target changes, we designed three steps to simulate the change of target behavior. As shown in Figure 5, both systems use the same method for object detection and tracking, thus, we skip the comparison of those parts, and the following three steps are designed for customer behavior recognition experiments.

#### 4.3.1. Step 1. Recognize Six Types of Behavior

At the beginning of building a CAR system, we select six common behavior definitions from existing researches for recognition, these definitions are described in Table 3. In Table 3, the symbol “A” refers to product A. For existing methods, these six types of behavior should be annotated to train models for recognition. As observed, in our proposed method, only four events (highlighted in bold font in Table 3) are required to define these behavior. The other events can be regarded as the union set of these four events.

#### 4.3.2. Step 2. Add New Behavior “Selecting”

Since the six types of behavior in step 1 cannot reveal the period of selecting products in the shelf area, we would like to recognize a new behavior named “selecting” to reveal more details in the video. “selecting” means that a customer is choosing products without making any picking decision. To add the recognition of a new type of behavior, E2E models should be rebuilt and new training data are required to train the models entirely. However, in the case of our proposed hierarchy, we only add a definition of “selecting” to step 2 in Table 3. The definition is explained as the whole person’s region moves in the shelf area. It is worth mentioning that the hand is usually occluded by the shelf, therefore, we use the whole person’s region (including arms) in the definition.

#### 4.3.3. Step 3. Discriminate the Behavior “Selecting”

As a person picks something by one hand or both hands could reflect potential things more than a summarized behavior “selecting”, we would like to find more details by discriminating “selecting”. Similarly to step 2, E2E models require time-consuming data collection and re-training. For the proposed hierarchy, we only redefine “selecting” in step 3 of Table 3. Selecting by one hand means a hand can be found out of the shelf. On the other hand, if there is no hand out of the shelf, the customer should be selecting by both hands. The three steps try to simulate the ever-changing needs. In this case, it needs to acquire more and more details from the video.

Since there is no annotation about the defined behavior of three steps in DS 1, we annotate DS 1 with the defined new behavior by modifying its original annotations. Since different types of behavior are required in the three steps, the number of annotated behavior varies during the three steps. DS 1 contains 6201, 7738, 7844 behavior annotations in step 1, step 2, step 3. DS 2 includes 153, 178, 181 behavior annotations in step 1, step 2, step 3. Since both datasets built different retail environments, we run both systems on both datasets. For the training of E2E models, we randomly choose 80% and 20% of the dataset as training set and test set. Table 4 shows the performance results of running both systems once. mAP refers to the mean average precision of customer behavior recognition. CPU/GPU implies the memory usage when running the system. FPS represents the running speed of the system. Results show that the E2E model-based system has a better recognition accuracy. However, the E2E model-based system uses more CPU/GPU memory and runs slower.

Both systems share a relatively low mAP on DS 1 mainly because of three reasons. First, DS 1 has a larger size than DS 2. Therefore, the models in both systems still need to be improved to fit a larger dataset. Second, the detection model has a bad detection accuracy on various types of products, which leads to the wrong input information for the higher level. Third, the fish-eye view caused by the camera distortion in DS 1 leads to a coordinate system that is more difficult to process compared to the direct top-view in DS 2. Nevertheless, the models in corresponding levels in our proposed hierarchy-based system can be updated to solve these problems. However, the E2E model-based system should be entirely modified because of its tightly coupled design.

Since more and more behavior should be recognized from step 1 to step 3, the results of mAP and FPS decreases for both systems, and they also use more and more memory. Though the hierarchy-based system does not outperform E2E model-based system on mAP, the small difference of mAP implies that the proposed hierarchy-based system is feasible. Additionally, the proposed system increases the running speed and saves memory usage, which indicates better efficiency.

### 4.4. Flexibility Evaluation

We design the three steps and prepare two datasets not only to evaluate the running performance, but also to evaluate both systems ability of fitting changes of demands and environments, which is called “flexibility”. For the E2E model-based system, each kind of change requires the time-consuming re-training of E2E models. To prepare the training data for the E2E models, we spent about two months annotating behavior in the DS 1 and DS 2. However, for the hierarchy based system, the behavior are defined manually by events. Thus, no training data are required for behavior recognition. To fit the three steps, namely the changes of demands, we only spent one day defining the new behavior with events. To adapt to the changes of dataset and environment, we also spent one day modifying the parameters ($threshold_{0}$, $threshold_{1}$, areas, $threshold_{2}$, and $threshold_{3}$ in Section 3.4) in level 3 where the events are recognized.

To quantify the amount of work during of a CAR system during fitting the changes of demands or environments, we calculate the flexibility with reference to the penalty of change in [36] as below:

(1) $$Flexibility=\frac{1}{N\cdot P_{n}},$$

where *N* is the number of videos in the dataset, $P_{n}$ is the cost of annotating each video. For the E2E model-based system, we should annotate new behavior in the whole dataset for model training. Therefore, $N_{E2E}=N$, $Flexibility_{E2E}=\frac{1}{N_{E2E}\cdot P_{n}}$. For the hierarchy-based system, we need to define new behavior by events, namely annotate new behavior by events. If we annotate one behavior in the video, we consider it defining the behavior by events because we recognize the events in our brain at first and then combine them to recognize the behavior. In DS 1 and DS 2, each video contains all types of behavior. Therefore, if we annotate all behavior in one video, it is considered as defining all new behavior by events. $N_{hie}=1$, $Flexibility_{hie}=\frac{1}{N_{hie}\cdot P_{n}}$. Thus,

(2) $$\frac{Flexibility_{hie}}{Flexibility_{E2E}}=\frac{\frac{1}{N_{hie}\cdot P_{n}}}{\frac{1}{N_{E2E}\cdot P_{n}}}=\frac{N_{E2E}}{N_{hie}}=\frac{N}{1}=N,$$

Equation (2) indicates that our proposed hierarchy-based system has the flexibility *N* times better than the E2E model-based system, where N=106 for DS 1 and N=19 for DS 2. This result shows that compared to the time-consuming re-training models, the proposed hierarchy-based system is malleable enough to adapt to the changes of demands and environments.

### 4.5. Structural Evaluation

Except for the experiment that runs two systems, we design a score to evaluate a system’s structure. The score is calculated from coupling, cohesion, and complexity [37], since these metrics reveal whether a system is efficient and easy to update. To calculate these metrics, firstly, we need to define the modules to be measure (i.e., the elementary entity). In the proposed system, we consider each level as a module, while in the case of E2E systems, each model is regarded as a module.

Based on the proposed formula in [37], we define the coupling as follows:

(3) $$Coupling=1-\frac{1}{\max(1,w+r)},$$

where *w* is the count of calling other module’s functions, *r* is the count of functions called by other modules. For the existing system, Figure 5 shows that Model 2 has similar functions of object detection and tracking as Model 1. Thus, w=1,r=0 for Model 2 and w=0,r=1 for Model 1. For the hierarchy, no levels share similar functions. Therefore, w=0,r=0 for each level. After calculating the coupling of each module, the coupling of the whole system is defined as the average of all modules’ coupling values.

In the case of cohesion, both systems are estimated to have a high cohesion in their modules, which means no difference in this metric. Therefore, the cohesion calculation is omitted in the evaluation.

Finally, for the complexity calculation, we use a modified version of [37], where some irrelevant values were not included, as described below:

(4) Complexity=IU+OU+MF,

where IU is the count of required inputs from the user, OU is the count of outputs from the system to the user, MF is the count of model files in the system. In our case, IU and OU indicate the count of data type instead of the amount of data. For instance, IU=1 for the existing system because the input is an image. IU=2 for the hierarchy because an extra input about the behavior definition is required. Finally, MF refers to the number of models in both systems.

To normalize the complexity value, the formula is modified as follows:

(5) $$Complexity^{\prime }=\frac{\left(IU-1)+(OU-1)+(MF-1\right)}{IU+OU+MF},$$

where $Complexity^{\prime }$ reaches the min value when IU=1, OU=1, MF=1, and $Complexity^{\prime }$ increases to a value no more than 1 when IU, OU, and MF increase. The complexity of the whole system is defined as the average of all modules’ complexity values.

Since a better system has lower coupling and complexity, we formulated the structure score (S) as

(6) $$S=\frac{1}{\alpha_{1}\cdot Coupling+\left(1-\alpha_{1}\right)\cdot Complexity^{\prime }},$$

where $\alpha_{1}\in \left[0,1\right]$, $\alpha_{1}$ is the weight of Coupling when designing a system, and a larger $\alpha_{1}$ means more emphasis on improving Coupling.

About the structure score, we set two parameters ($\alpha_{1},n$) to influence the score and observe its changes, where *n* is the number of implemented models in each system. For the existing system, Figure 5 shows a simple system with only two models. However, usually, more models are required in a common structure, such as Figure 1. Because it is difficult to recognize all customer behavior by only one model and it is the same for the other types of data. Therefore, Coupling and MF may change with the increasing number of models.

**Figure 8:**
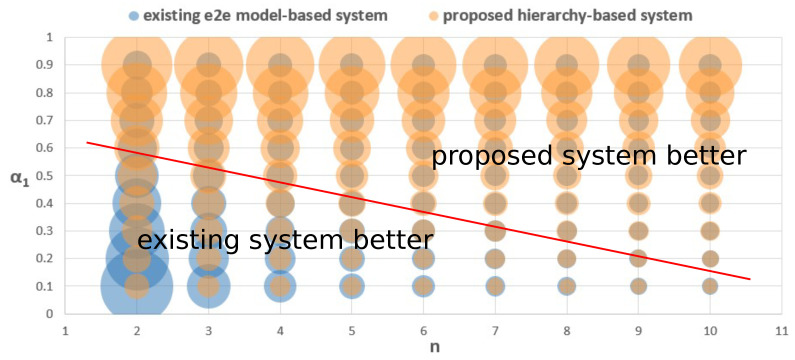
Structure scores of the E2E model-based system and the proposed hierarchy-based system.

Figure 8 shows the change of the structure score when modifying $\alpha_{1}$ and *n*. The x-axis is *n* and the y-axis is $\alpha_{1}$. The circle radius refers to the structure score. A larger circle represents a better structure score. The chart shows that our proposed system have a better score when $\alpha_{1}$ and *n* increase. In most cases, the proposed system has a better score. Since a better structure score implies high efficiency and maintainability, Figure 8 indicates that our proposed hierarchy-based system is better in most cases. To sum up, the performance, flexibility, and structure evaluation results show that the proposed hierarchy-based system has better adaptability, efficiency, and maintainability.

## 5. Conclusions

In this research, we proposed a hierarchy-based CAR system to recognize different types of CA in retail stores to fit the ever-changing target conditions. The existing E2E model-based CAR system cannot fit the changes because of its low efficiency, flexibility, and maintainability. Our proposed hierarchy-based CAR system contains a hierarchy that separates CAR tasks into four levels. Input data is processed level by level and each level outputs a type of CA. Additionally, level primitives provides a solution to malleable behavior recognition. Two comparison experiments are conducted to evaluate efficiency, flexibility, and maintainability of both systems. Results of running performance show that our proposed CAR system is feasible to conduct CAR tasks efficiently, the calculation of flexibility implies better flexibility of our proposed CAR system, and the structure score of both systems indicates that our proposed CAR system is easier to maintain. Despite the limited accuracy results, since the method in each level can be replaced by any better one, more suitable methods are expected to be applied to corresponding level to improve the accuracy in the future work. Furthermore, nearly all researches focus on human–product behavior recognition currently because of the complex variation in human–human behavior. With the malleable behavior recognition method in this paper, human–human behavior classification is expected to be realized in the future. Besides, though the design of hierarchy is limited to the retail environment, we expect that the hierarchical structure could be applied to the general behavior recognition.

## Figures and Tables

**Table 1:** Categories of target CA types in the existing CAR systems.

Categories	CA Types	References
Object Location	Customer Location	[3,4,5,6,7,8,9,10,11,12,13,14,15,16,17,18,19,20]
	Customer Location (Body Parts)	[1,3,4,21]
	Other Object Location (Products, Baskets, Carts)	[1,22,23]
Object Movement	Object Motion	[24,25]
	Object Trajectory	[3,15,16,17,18,19,22,23,24]
Customer Behavior	Passing by a Shelf	[3,4,15]
	Turning to a Shelf	[3]
	Viewing a Shelf	[3,19]
	Touching a Shelf	[3,4,16,25]
	Picking up a Product from a Shelf	[3,4,15,16,24,25]
	Putting a Product back to a Shelf	[3,4,15,16]
	Putting a Product into a Basket/Cart	[3]
	Holding a Product	[19]
	Browsing a Product in a Hand	[19,25]
	Trying on Clothes	[25]

**Table 2:** Example definition of the behavior “pick a product”.

Behavior	Event Pattern	Content of Events (* = Any Value)
Pick a product	ME 1 → ME 2 & RE 3	ME 1: [hand], [towards shelf], [* → SA]
		ME 2: [hand], [leave shelf], [SA → *]
		RE 3: [product A], [following], [*], [hand]

**Table 3:** Behavior definitions of the performance evaluation.

Step	Behavior	Event Pattern	Content of Events (* = Any Value)
	Walking: walking in VA	ME 1	**ME 1**: [person], not [stop], [VA → VA]
	Viewing:	ME 1	ME 1: [person], [stop], [VA → VA]
	stop and view products		
	Browse:	ME 1 & RE 2	ME 1: [person], [*], [VA → VA]
	browse a product on hands		**RE 2**: [A], [following], [*], [hand]
	Pick:	ME 1 → ME 2 & RE 3	**ME 1**: [hand], [towards shelf], [* → SA]
Step 1	pick a product out of shelf		**ME 2**: [hand], [leave shelf], [SA → *]
			RE 3: [A], [following], [*], [hand]
	Touch:	ME 1 → ME 2	ME 1: [hand], [towards shelf], [* → SA]
	pick nothing out of shelf		ME 2: [hand], [leave shelf], [SA → *]
	Return:	ME 1 & RE 2 → ME 3	ME 1: [hand], [towards shelf], [* → SA]
	return a product to shelf		RE 2: [A], [following], [*], [hand]
			ME 3: [hand], [leave shelf], [SA → *]
Step 2	Selecting: select products in SA	ME 1	ME 1: [person], [*], [* → SA]
Step 3	Select by one hand	ME 1 & ME 2	ME 1: [person], [*], [* → SA]
		ME 2: [hand], [*], [VA → VA]
	Select by both hands	ME 1	ME 1: [person], [*], [* → SA]

**Table 4:** Performance results of both CAR systems on DS 1 and DS 2.

Step	System Type	DS 1				DS 2			
		mAP	CPU	GPU	FPS	mAP	CPU	GPU	FPS
Step 1	hierarchy-based	77.82%	1.8 GB	4.9 GB	17	94.16%	1.8 GB	4.9 GB	17
	E2E model-based	82.68%	2.4 GB	5.0 GB	13	94.30%	2.4 GB	5.0 GB	13
Step 2	hierarchy-based	74.36%	1.9 GB	4.9 GB	15	91.78%	1.9 GB	4.9 GB	15
	E2E model-based	81.27%	2.5 GB	5.0 GB	11	93.94%	2.5 GB	5.0 GB	11
Step 3	hierarchy-based	73.77%	1.9 GB	4.9 GB	13	90.37%	1.9 GB	4.9 GB	13
	E2E model-based	80.74%	2.5 GB	5.1 GB	10	92.16%	2.5 GB	5.1 GB	10

## Data Availability

Not applicable.

## Abbreviations

The following abbreviations are used in this manuscript:

CA	Customer Activity
CAR	Customer Activity Recognition
E2E	End-to-End
HOG	Histogram of Oriented Gradients
CNN	Convolutional Neural Network
UWB	Ultra-Wideband
IoU	Intersection over Union
HMM	Hidden Markov Model
LSTM	Long-short Term Memory
SVM	Support Vector Machine
FPS	Frame Per Second
ATP	Approximate Trajectory Partitioning
ME	Motion Event
RE	Relation Event
SA	Shelf Area
VA	Viewing Area
